# Stigma, Food Insecurity, and Limited Social Support as Psychosocial Correlates of Antiretroviral Therapy Adherence Among Pregnant Women Living With HIV: Cross-Sectional Study

**DOI:** 10.2196/84914

**Published:** 2026-04-27

**Authors:** Setor K Sorkpor, Ibrahim Yigit, Rachel G A Thompson, Brittany L Lane, Geoffrey Anguyo, Jerry John Ouner

**Affiliations:** 1 College of Nursing, Brain Science and Symptom Management Center, Florida State University, Tallahassee, FL, United States; 2 College of Nursing, Florida State University, Tallahassee, FL, United States; 3 University of Ghana, Africa Interdisciplinary Research Institute, University of California San Francisco, 1975 4th St, San Francisco, CA, 94158, United States, 1 415-353-3000; 4 Kigezi Healthcare Foundation, Mbarara University of Science and Technology, Kabale, Uganda; 5 Department of Family Health Care Nursing, University of California San Francisco, San Francisco, CA, United States

**Keywords:** prenatal women, sub-Saharan Africa, discrimination, internalized stigma, interpersonal support, food scarcity, psychosocial factors

## Abstract

**Background:**

Adherence to antiretroviral therapy (ART) during pregnancy is critical for maternal health and the prevention of vertical HIV transmission. In Uganda, where HIV prevalence remains high, pregnant women living with HIV face intersecting structural and psychosocial challenges, including stigma, food insecurity, and limited social support. Although each factor has been linked to ART nonadherence, less is known about how these factors co-occur within individuals and jointly shape vulnerability to nonadherence during pregnancy.

**Objective:**

This study used latent profile analysis to identify empirically derived psychosocial vulnerability profiles reflecting the co-occurrence of stigma, food insecurity, and limited social support among pregnant women living with HIV in Uganda and examine whether profile membership is associated with ART adherence.

**Methods:**

We conducted a cross-sectional analysis of 167 pregnant women living with HIV recruited from 6 health facilities in Uganda between June and December 2020. Measures included experienced HIV stigma, internalized HIV stigma, household food insecurity, and interpersonal social support. Latent profile analysis identified psychosocial profiles, and linear regression models assessed associations between profile membership and ART adherence, adjusting for sociodemographic covariates.

**Results:**

A 2-class solution provided the best model fit (entropy=0.93). The higher-risk profile (75/167, 45.1%) was characterized by increased experienced stigma (mean score 1.97, SE 0.05), higher internalized stigma (mean score 2.66, SE 0.07), severe food insecurity (mean score 7.53, SE 0.20), and lower social support (mean score 2.06, SE 0.04). The lower-risk profile (92/167, 54.9%) showed significantly lower internalized stigma (mean score 2.32, SE 0.04; *P*<.001), lower experienced stigma (mean score 1.72, SE 0.05; *P*<.001), minimal food insecurity (mean score 0.82, SE 0.15; *P*<.001), and slightly higher social support (mean score 2.14, SE 0.04; *P*<.001). Membership in the higher-risk profile was associated with significantly lower ART adherence compared with membership in the lower-risk profile (B=0.88; β=0.40, 95% CI 0.02-0.78; *P*=.04).

**Conclusions:**

Distinct psychosocial profiles are meaningfully associated with ART adherence among pregnant women living with HIV in Uganda. By moving beyond single-risk models, these findings demonstrate the added value of person-centered analytic approaches for identifying subgroups of pregnant women living with HIV exposed to clustered psychosocial and structural vulnerabilities. The findings underscore the need for multicomponent, person-centered interventions that simultaneously address stigma, food insecurity, and limited social support rather than treating these challenges in isolation. Identifying empirically derived vulnerability profiles provides a targeted framework for prioritizing high-risk subgroups and informing contextually appropriate intervention design. Addressing these intersecting vulnerabilities is essential for improving maternal ART adherence and supporting efforts to prevent vertical HIV transmission in high-burden settings.

## Introduction

Although HIV prevention and treatment coverage have expanded across sub-Saharan Africa (SSA), Uganda continues to shoulder a high HIV burden [1,2]. An estimated 1.5 million Ugandans live with HIV, 57% of whom are women [1]. Against this backdrop, pregnancy and the early postpartum period are particularly consequential for maternal health and prevention of vertical transmission [3,4]. Accordingly, consistent antiretroviral therapy (ART) adherence during this window is essential [4-6]. Uganda has adopted HIV treatment for all, a World Health Organization strategy that provides lifelong ART to all people living with HIV, including pregnant and breastfeeding women living with HIV regardless of clinical stage or CD4 T-lymphocyte count [6-9]. However, nonadherence remains common, particularly during the postpartum period. Evidence from Uganda and SSA indicates that up to one-third of women miss doses of ART, thereby increasing the risk of virologic failure and mother-to-child transmission [6,10-12].

In this context, a complex array of structural and psychosocial factors undermines ART adherence among pregnant and postpartum women living with HIV in Uganda [10]. HIV-related stigma remains one of the most pervasive barriers to sustained engagement in care [1,13,14]. Stigma operates at multiple levels: externalized stigma (discrimination, rejection, and social exclusion by others) and internalized stigma (self-directed shame, guilt, and diminished self-worth) [15,16]. Women living with HIV frequently report experiencing stigma from family members, community figures, and health care providers, which discourages disclosure and contributes to social withdrawal and disengagement from care [17,18]. Internalized stigma is also associated with increased depressive symptoms and reduced treatment self-efficacy among people entering HIV care, factors that can undermine ART adherence during pregnancy and the early postpartum period [19-21].

Beyond stigma, household food insecurity is another critical, often underappreciated barrier to optimal ART adherence among pregnant and postpartum women living with HIV. Recent phone survey data show that 48% of Ugandan households experience moderate food insecurity and 11% experience severe food insecurity, with the highest burden in rural areas [22]. For women managing HIV during pregnancy, food insecurity magnifies the challenges of chronic illness by intensifying common ART side effects such as nausea, dizziness, and gastrointestinal discomfort when medications are taken without food [23,24]. These amplified side effects discourage adherence, increase depressive symptom severity, and compromise viral suppression [25,26]. Food insecurity also heightens psychological stress, limits care seeking, and undermines social participation and economic independence, thereby reinforcing HIV-related stigma [25,27]. Collectively, these pathways elevate the risk of suboptimal adherence during pregnancy and the early postpartum period.

Importantly, interpersonal social support can buffer the harmful effects of stigma and food insecurity on HIV-related outcomes. Longitudinal studies in rural Uganda and western Kenya demonstrate that emotional and instrumental support reduce the impact of food insecurity on depression and improve ART adherence [28,29]. Peer-based interventions, including peer mother counseling and online support groups, have also shown promise in improving adherence and disclosure among pregnant and postpartum women living with HIV in Uganda [30,31]. However, such support is not universally accessible. Qualitative research has documented that many women experience relationship conflict, lack of disclosure, and rejection following an HIV diagnosis, which limits their access to emotional or practical assistance [17,32].

Despite this evidence, while stigma, food insecurity, and social support have each been studied individually as predictors of ART adherence [33-35], their co-occurrence and interaction remain poorly understood among pregnant and postpartum women in SSA [36,37]. Traditional variable-centered approaches such as early regression models [38] often examine these predictors in isolation and may overlook how multiple adversities interact to shape adherence outcomes [39]. Syndemic frameworks emphasize the synergistic clustering of social and structural challenges, which may amplify risk beyond the sum of individual effects [39,40]. Person-centered methods such as latent profile analysis (LPA) offer a rigorous approach to identifying subgroups of individuals who share similar constellations of psychosocial characteristics [41].

Accordingly, LPA has been increasingly applied in HIV research to detect syndemic vulnerability profiles and adherence-related behavioral patterns among adults in diverse contexts, including SSA [42-44]. However, its use among pregnant and postpartum women remains limited, particularly in high-burden, resource-constrained settings. Applying LPA in this context may uncover subgroups of women disproportionately affected by overlapping stressors and inform more targeted, tailored interventions.

This study addresses this gap by applying LPA to identify vulnerability subgroups among pregnant women living with HIV in Uganda based on 4 theoretically and empirically grounded indicators: experienced HIV stigma, internalized HIV stigma, household food insecurity, and perceived interpersonal social support. We then examined whether profile membership was associated with ART adherence adjusting for sociodemographic covariates. We hypothesized that members of higher-risk profiles, characterized by greater stigma and food insecurity combined with lower levels of support, would report significantly lower ART adherence compared with members of lower-risk profiles. By identifying distinct constellations of co-occurring risk factors, this study aimed to inform more targeted and contextually appropriate interventions to improve maternal adherence and advance Uganda’s progress toward achieving its 95-95-95 targets.

## Methods

### Data, Participants, and Procedures

Data were drawn from a larger longitudinal study examining factors that influence retention in HIV care among pregnant and postpartum women in Uganda. Details of the parent study design and data collection procedures have been reported elsewhere [45]. Participants were recruited from 6 participating health care facilities through a clinic-based recruitment process. Midwives and nurses involved in routine antenatal and HIV care served as initial points of contact and systematically identified potentially eligible women during routine clinic visits. Eligibility criteria included living with HIV, being in the third trimester of pregnancy, and enrollment in an ART or prevention of mother-to-child transmission program.

Women who met the eligibility criteria and expressed interest were referred to trained study staff, who provided a detailed explanation of the study, confirmed eligibility, obtained written informed consent, and administered baseline questionnaires. Baseline data collection took place between June and August 2020. Approximately 3 months later, participants were recontacted to complete follow-up assessments, including additional sociodemographic and ART adherence–related measures, between October and December 2020. Recruitment was intentionally anchored in late pregnancy, when women are approaching delivery and facing heightened clinical, psychosocial, and structural demands, to facilitate follow-up into the early postpartum period in the parent longitudinal study.

This analysis used baseline data only and was conducted as a cross-sectional study. Baseline assessment during the third trimester provided a uniform and clinically salient time point, capturing psychosocial and structural vulnerabilities during a period of heightened relevance for ART adherence and prevention of vertical HIV transmission. Restricting analyses to baseline data minimized heterogeneity related to postpartum transitions and allowed for the identification of contemporaneous psychosocial vulnerability profiles prior to delivery. Longitudinal findings from the parent study examining postpartum outcomes have been reported previously [45].

### Ethical Considerations

This study was conducted in accordance with the principles of the Declaration of Helsinki and was reviewed and approved by the Institutional Review Board of the University of California, San Francisco (approval 20-30261), and the Mbarara University of Science and Technology Research Ethics Committee (approval MUREC 1/7). Written informed consent was obtained from all participants prior to enrollment. Participants did not receive compensation for their participation. Participant confidentiality was maintained throughout the study, and all data were deidentified prior to analysis and stored securely, with access restricted to authorized study personnel. This analysis used data from an approved parent study, and all ethics approvals and consent procedures were conducted as part of the original study protocol [45].

### Measures

#### Overview

All study measures were administered by trained research staff with experience working with pregnant women living with HIV in Uganda. Instruments were selected based on prior use in HIV research and conceptual relevance to the study objectives. The measures were reviewed and piloted prior to data collection to ensure clarity and contextual appropriateness for the study population. Participants were provided with assistance as needed during questionnaire administration to support comprehension.

#### Internalized HIV Stigma

Internalized HIV stigma was assessed using the Internalized Stigma of HIV/AIDS Tool [46], a 10-item measure previously used in HIV research, including studies conducted in SSA, in which respondents rate each statement on a 5-point Likert scale ranging from “strongly disagree” to “strongly agree.” Items prompt participants to reflect on their self-perceptions and feelings since receiving an HIV diagnosis. Example items include “I feel blemished” and “I feel ashamed about having HIV/AIDS.” We calculated mean scores, with higher scores indicating greater internalized HIV stigma. In this sample, the scale demonstrated good internal consistency (Cronbach α=0.81).

#### Experienced HIV Stigma

Experienced HIV stigma was assessed using the Externalized Stigma of HIV/AIDS Tool (ESAT), a 10-item measure capturing participants’ experiences of stigma and discrimination from others in response to their HIV status. The ESAT was adapted from the Internalized Stigma of AIDS Tool to capture enacted or experienced HIV-related stigma, which is conceptually distinct from internalized stigma. Participants rate each statement on a 5-point Likert scale ranging from “strongly disagree” to “strongly agree.” Example items include “I have been excluded from social events” and “I have been verbally abused or ridiculed.” Average scores were calculated, with higher scores indicating greater experienced HIV stigma. In this sample, the scale demonstrated good internal consistency (Cronbach α=0.74). The full list of ESAT items is provided in Multimedia Appendix 1.

#### Household Food Insecurity

Household food insecurity was measured using items from the Household Food Insecurity Access Scale (HFIAS) [47], which assesses household food access over the previous 30 days. The HFIAS includes paired items that first assess the presence of specific food insecurity conditions (“yes” or “no”) and, if endorsed, follow with questions assessing frequency. In this analysis, only the dichotomous HFIAS items assessing the presence of food insecurity conditions were used. The corresponding frequency follow-up items were not included because participants who responded “no” to the initial presence questions did not provide frequency data, resulting in substantial missingness on the frequency items. Dichotomous items were coded as 0 (“no”) or 1 (“yes”) and summed to create a composite score reflecting the number of food insecurity conditions endorsed, with higher scores indicating greater household food insecurity. Internal consistency for this composite was high (Cronbach α=0.96)

#### Interpersonal Social Support

Perceived interpersonal social support was assessed using the Interpersonal Support Evaluation List–12 [48], a widely used and psychometrically validated measure of perceived availability of social resources. The Interpersonal Support Evaluation List–12 consists of 12 items rated on a 4-point Likert scale (0=“definitely false”; 3=“definitely true”). Sample items include “If I were sick, I could easily find someone to help me with my daily chores” and “There is someone I can turn to for advice about handling problems with my family.” Item scores were averaged to yield a composite score, with higher scores indicating greater perceived interpersonal social support. In this study, the Cronbach α was 0.71, suggesting acceptable reliability.

#### ART Adherence

ART adherence was assessed using 3 items from the Center for Adherence Support Evaluation Adherence Index [49], which capture common adherence challenges. Participants responded to the following three items: (1) “How often do you feel that you have difficulty taking your HIV medications on time?” (response options: “never,” “rarely,” “most of the time,” and “all of the time”); (2) “On average, how many days per week would you say that you missed at least one dose of your HIV medications?” (response options: “every day,” “4‐6 days/week,” “2-3 days/week,” “once a week,” “less than once a week,” and “never”); and (3) “When was the last time you missed at least one dose of your HIV medications?” (response options: “within the past week,” “1‐2 weeks ago,” “3‐4 weeks ago,” “between 1 and 3 months ago,” “more than 3 months ago,” and “never”). Responses across these 3 items were coded and summed to create an ART adherence composite score ranging from 3 to 16, with higher scores indicating more optimal adherence to ART. The Cronbach α was calculated as 0.76, suggesting good internal reliability.

### Data Analysis

Descriptive statistics were calculated for all study variables. LPA was conducted to identify unobserved subgroups (latent classes) of participants based on 4 continuous indicators: experienced HIV stigma, internalized HIV stigma, food insecurity, and interpersonal support. Model selection (the number of profiles) was guided by multiple fit indexes: the Akaike information criterion (AIC), Bayesian information criterion (BIC), consistent AIC, sample size–adjusted BIC, and classification likelihood criterion. Higher model fit was indicated by lower values of these indexes. Model interpretability and entropy values (ranging from 0 to 1) were also used to assess classification accuracy, with higher values indicating more precise assignment of individuals to latent classes. In determining the optimal number of classes, parsimony and fit indexes were balanced with conceptual clarity and class separation. Parameter estimates (means, variances, and SEs) for each class were extracted and compared across latent profiles to describe group characteristics.

Next, we conducted a linear regression analysis to examine the association between the latent classes and optimal ART adherence adjusting for age, educational status of both women living with HIV and their partners, religion, place of residence, partners’ HIV status, and perceived financial status. All independent variables, including covariates, were entered simultaneously into the model. All analyses were cross-sectional and performed using Jamovi (version 2.3) [50].

Because participants were recruited through clinic-based sampling rather than a probability-based survey design, no survey weights were applied in the analyses. The study sample was not intended to be nationally representative, and therefore, weighting procedures designed to correct for unequal selection probabilities or population-level inference were not appropriate. All analyses were conducted using unweighted data, consistent with prior clinic-based studies of pregnant women living with HIV in similar settings. The analytic focus was on identifying within-sample psychosocial vulnerability profiles rather than producing population-level prevalence estimates.

## Results

### Descriptive Statistics

A total of 167 pregnant women living with HIV were included in the analysis. As shown in Table 1, the mean age was 28.01 (SD 6.14; range 17-45) years. Most participants (124/167, 74.3%) reported being in a relationship and living with a partner. Regarding educational level, most (118/167, 70.7%) indicated primary school education as their highest level. When asked about their partners’ educational level, 47.0% (77/164) reported primary school education, whereas 19.5% (32/164) indicated that they did not know. Most participants (96/166, 57.8%) identified as Protestant, and most (141/166, 84.9%) lived in rural areas. Slightly more than half (84/160, 52.5%) reported that their partner was HIV positive, whereas others reported that their partner was HIV negative (46/160, 28.7%) or that they did not know their partner’s status (30/160, 18.8%). Finally, most participants (139/166, 83.7%) described their financial situation as poor or not enough. Mean scores were 1.84 (SD 0.47; range 1.00‐4.20) for experienced stigma, 2.48 (SD 0.54; range 1.40‐3.80) for internalized stigma, 3.88 (SD 3.65; range 0‐9) for food insecurity, 2.11 (SD 0.38; range 0.75‐3.00) for interpersonal social support, and 14.5 (SD 2.31; range 7‐16) for ART adherence.

**Table 1.** Descriptive statistics.

Variable	Values
Current relationship status (n=167), n (%)	
Widowed	3 (1.8)
Divorced	8 (4.8)
In a relationship—living with partner	124 (74.3)
In a relationship—not living with partner	27 (16.2)
Single, never married, or no current partner	5 (3.0)
Educational level—participant (n=167), n (%)	
Never been to school	14 (8.4)
Primary school	118 (70.7)
Secondary school (O-Level)	28 (16.8)
Higher secondary school (A-Level)	4 (2.4)
University degree or higher	3 (1.8)
Educational level—partner (n=164), n (%)	
Never been to school	9 (5.5)
Primary school	77 (47.0)
Secondary school (O-Level)	31 (18.9)
Higher secondary school (A-Level)	4 (2.4)
Diploma	5 (3.0)
University degree or higher	6 (3.7)
Did not know	32 (19.5)
Religion (n=166), n (%)	
Protestant	96 (57.8)
Catholic	54 (32.5)
Born-again Christian	7 (4.2)
Muslim	9 (5.4)
Place of residence (n=166), n (%)	
Rural	141 (84.9)
Urban	25 (15.1)
Partner HIV status (n=160), n (%)	
Positive	84 (52.5)
Negative	46 (28.7)
Did not know	30 (18.8)
Financial situation (n=166), n (%)	
Poor or not enough	139 (83.7)
Enough	27 (16.3)
Experienced HIV stigma score, mean (SD; range: possible range)	1.84 (0.47; 1.00-4.20; 1-5)
Internalized HIV stigma score, mean (SD; range; possible range)	2.48 (0.54; 1.40-3.80; 1-5)
Food insecurity score, mean (SD; range; possible range)	3.88 (3.65; 0-9; 0-9)
Interpersonal social support score, mean (SD; range; possible range)	2.11 (0.38; 0.75-3.00; 0-3)
ART^a^ adherence score mean (SD; range; possible range)	14.5 (2.31; 7-16; 3-16)

a ART: antiretroviral therapy.

### LPA Results

LPA was conducted to identify distinct subgroups of participants based on their scores on the selected indicators (ie, experienced HIV stigma, internalized HIV stigma, food insecurity, and interpersonal support). Competing models with 2 and 3 latent classes were compared. The results suggest that model fit indexes favored the 2-class solution over the 3-class solution. Specifically, the 2-class model had the lowest values for the AIC (1408), BIC (1449), consistent AIC (1462), and sample size–adjusted BIC (1407), all indicating better fit. The 2-class model also exhibited higher entropy (0.93) compared with the 3-class model (0.82), suggesting more accurate classification of individuals into latent classes. Although the 3-class model had slightly better classification likelihood (classification likelihood criterion=1411 vs 1384 for the 2-class model), this was not sufficient to outweigh the superior overall fit of the 2-class solution. Taken together, these criteria support the 2-class model as the most parsimonious and best-fitting solution.

**Figure 1.**
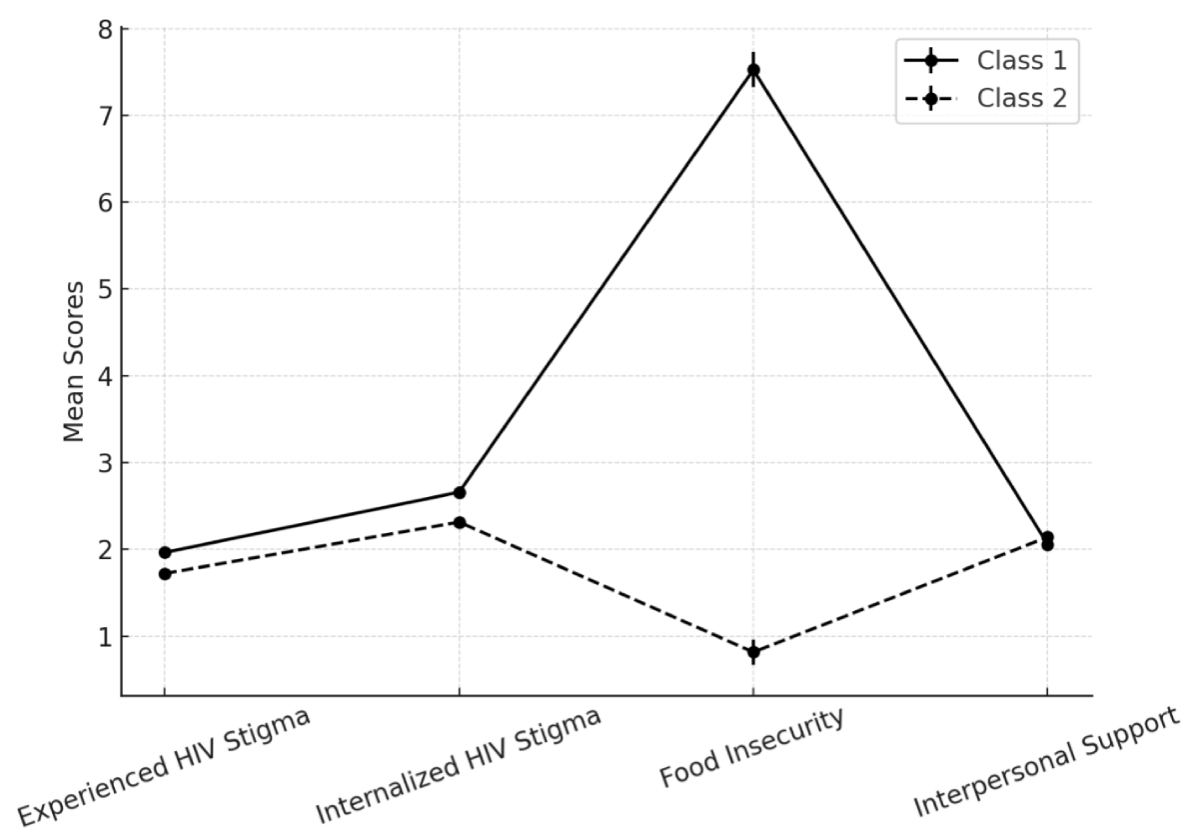
Profile plot of latent classes.

As shown in Figure 1, there were 2 latent classes that emerged. Class 1 (higher-risk profile; 75/167, 45.1%) was characterized by greater experienced HIV stigma (mean 1.97; SE 0.05), higher internalized HIV stigma (mean 2.66; SE 0.07), high food insecurity (mean 7.53; SE 0.20), and lower interpersonal support (mean 2.06; SE 0.04). In contrast, class 2 (lower-risk profile; 92/167, 54.9%) showed slightly lower experienced HIV stigma (mean 1.72; SE 0.04), lower internalized HIV stigma (mean 2.32; SE 0.05), much lower food insecurity (mean 0.82; SE 0.15), and slightly higher interpersonal support (mean 2.14; SE 0.04). All differences between classes were statistically significant (*P*<.001). These findings indicate 2 interpretable and distinct profiles of pregnant women living with HIV in Uganda, differing substantially in structural and psychosocial vulnerability.

### Linear Regression Analysis

A linear regression analysis was conducted to examine the association between latent profile membership (lower-risk profile vs higher-risk profile) and optimal ART adherence while adjusting for the covariates. Latent class membership emerged as a significant predictor of ART adherence. Specifically, compared with women living with HIV in the lower-risk profile (class 2), those in the higher-risk profile (class 1) reported significantly lower levels of optimal ART adherence (*B*=0.88; β=0.40, 95% CI 0.02-0.78; *t*_133_=2.07; *P*=.04). These results suggest that latent profiles capturing structural and psychosocial vulnerabilities are meaningfully associated with ART adherence above and beyond key demographic and contextual factors.

## Discussion

### Principal Findings

This study aimed to identify psychosocial vulnerability profiles among pregnant women living with HIV in Uganda and examine their associations with ART adherence during pregnancy. Consistent with these objectives, we identified 2 empirically distinct vulnerability profiles that differed significantly in levels of HIV stigma, food insecurity, and interpersonal social support and were significantly associated with ART adherence. Women living with HIV in the higher-risk profile, characterized by higher experienced and internalized HIV stigma, severe food insecurity, and lower interpersonal support, reported significantly lower ART adherence relative to their lower-risk counterparts. The converging of HIV stigma and food insecurity within the high-risk group suggests a pattern of intersecting stressors that may amplify psychological distress, impair medication adherence, and disrupt care engagement during the perinatal period.

These results align with syndemic theory, which posits that co-occurring adversities can interact synergistically to undermine health behaviors [39,40]. Unlike prior variable-centered studies, our LPA demonstrates how stigma, food insecurity, and social support co-occur to shape distinct vulnerability profiles. Our findings are consistent with those of prior Ugandan studies showing that stigma undermines ART adherence [51], food insecurity disrupts treatment consistency [52], and social support facilitates adherence [53]. Similar associations have been reported in neighboring countries, where stigma [54], food insecurity [55], and family support [56] have been identified as critical determinants of adherence. Our study extends this literature by highlighting how these factors co-occur within subgroups of pregnant women living with HIV, thereby highlighting heterogeneity in vulnerability that is obscured when determinants are examined individually. By situating our results within this evidence, we not only show alignment with prior work but also advance understanding by demonstrating the buffering role of interpersonal support, a dimension often overlooked in previous analyses. This person-centered perspective provides a more nuanced understanding of vulnerability among pregnant women living with HIV and complements prior studies that have examined these factors in isolation.

Our findings also underscore the need for interventions that address intersecting structural and psychosocial vulnerabilities identified in the higher-risk profile. Multicomponent approaches that respond to both material needs and social stressors are likely to be most effective in this context. In Uganda, the Friends for Life Circles peer group program combined peer mother mentoring, community ART distribution, and income-generating support, resulting in improved postpartum retention and engagement in care [57]. The program’s design allowed peer mothers to provide psychosocial support and role modeling, whereas community-based ART delivery reduced barriers such as transport costs and clinic waiting times. The inclusion of income-generating activities further addressed underlying economic vulnerability, helping women sustain care engagement [57]. In Kenya, the Shamba Maisha [58,59] livelihood intervention provided small loans, farming tools, and agricultural and financial training to people living with HIV. Participants reported reduced internalized stigma and improved psychosocial well-being, attributed to increased economic productivity and a regained sense of social value [58,59]. By improving household food security and restoring participants’ economic agency, the intervention not only strengthened material conditions but also enhanced social standing within the community [58,59].

Together, these examples highlight the role of economic empowerment in creating conditions that enable ART adherence and demonstrate the value of integrated, context-sensitive strategies that simultaneously address food insecurity, HIV stigma, and limited social support. Building on this evidence, future HIV interventions in Uganda should consider integrating community-based delivery models such as mentor mothers and community health workers with nutritional assistance. Potential strategies include food vouchers to meet immediate dietary needs and agricultural subsidies to support sustainable household food production, particularly in rural communities where subsistence farming is common. Conditional food support linked to engagement in antenatal HIV care may further help disrupt the cycle of poverty, stigma, and disengagement from care among pregnant women living with HIV.

Food insecurity may exacerbate stigma-related stress through multiple interacting pathways. Physiologically, taking ART without sufficient food intake has been linked to intensified side effects, which may undermine treatment adherence and persistence [60]. Psychologically, food insecurity contributes to depressive symptoms and reduced self-efficacy, particularly in rural Ugandan settings where poverty and HIV stigma are closely intertwined [25]. Interpersonally, social support serves a protective function by providing emotional reassurance and practical assistance, including help with medication adherence, clinic attendance, and disclosure [61]. Our findings align with these mechanisms: participants in the lower-risk latent profile reported higher levels of perceived support, suggesting that even modest increases in interpersonal support may buffer the adverse effects of structural and psychosocial stressors. This interpretation is further supported by meta-analytic evidence indicating that social support is a consistent facilitator of ART adherence among pregnant and breastfeeding women across SSA [62,63].

Although this study advances the HIV literature by applying a person-centered analytic approach during a critical perinatal period and integrating both structural and psychosocial determinants of ART adherence, several limitations should be noted. First, although the data were drawn from a longitudinal cohort, this analysis was cross-sectional. This limits causal inference, making it unclear whether stigma, food insecurity, and social support influence ART adherence or whether nonadherence may exacerbate these vulnerabilities. Future studies should use longitudinal or implementation science designs to clarify temporal and causal relationships. Second, ART adherence was assessed via self-report, which may be subject to recall and social desirability biases. Incorporating objective measures such as electronic dose monitoring or pharmacy refill records should strengthen adherence measurement in future research. Third, our analysis did not include other relevant determinants of adherence, such as depressive symptoms, intimate partner violence, or transportation barriers, which may further refine vulnerability profiles. Fourth, because the study sample was relatively small and recruited from rural health facilities, the findings may not be generalizable to urban populations or other regions. Replication with larger and more diverse samples is warranted to strengthen external validity. Future research should also assess whether additional profiles emerge in larger and more diverse samples.

Although the data were collected in 2020, the structural and psychosocial challenges examined in this study, including HIV-related stigma, food insecurity, and limited social support, remain persistent barriers to ART adherence among pregnant women in Uganda and other high-burden settings. In addition, shifts in the global HIV funding landscape toward more constrained and efficiency-focused models heighten the importance of person-centered approaches that can identify and prioritize subgroups facing clustered vulnerabilities, thereby supporting more targeted and sustainable intervention strategies.

### Conclusions

This study highlights the need to urgently address intersecting psychosocial and structural challenges that compromise ART adherence among pregnant women living with HIV. Identifying latent vulnerability profiles offers a targeted framework for directing limited resources to those at greatest risk of disengagement. Intervention strategies should be grounded in the lived experiences of pregnant women living with HIV and must address overlapping adversities rather than treating risks in isolation. Integrated approaches that combine HIV stigma reduction, nutritional support, and strengthened social networks are essential for efforts to promote maternal health and prevent vertical HIV transmission. By demonstrating how these determinants cluster within identifiable subgroups, our findings underscore the value of person-centered methods for guiding intervention design. Addressing these vulnerabilities is essential not only for advancing Uganda’s progress toward the 95-95-95 targets but also for informing global strategies to improve maternal and child health outcomes in high-burden settings.

## Supplementary material

10.2196/84914 Multimedia Appendix 1 Questionnaires used in the study.

## Abbreviations

AIC: Akaike information criterion
ART: antiretroviral therapy
BIC: Bayesian information criterion
ESAT: Externalized Stigma of HIV/AIDS Tool
HFIAS: Household Food Insecurity Access Scale
LPA: latent profile analysis
SSA: sub-Saharan Africa
