# Supplementary material for: Stigma, Food Insecurity, and Limited Social Support as Psychosocial Correlates of Antiretroviral Therapy Adherence Among Pregnant Women Living With HIV: Cross-Sectional Study
Source: JMIR Public Health Surveill. 2026 Apr 27;12:e84914. doi: 10.2196/84914 (PMC13120754; doi:10.2196/84914)
Supplement: Multimedia Appendix 1 [file publichealth-v12-e84914-s001.docx]

Appendix 1  **ID # …………………….**

**Data Collection Tool**

**Questionnaire – English Language**

| We are conducting a survey on factors that influence postpartum retention in HIV care among HIV positive mothers in Kabale District. I will ask you some questions on this questionnaire. The questions usually take about 15 to 20 minutes. All of the answers you give will be confidential and will not be shared with anyone other than members of our survey team. Participation in the study is voluntary, but we hope you will agree to answer the questions since your views are important. If I ask you any question you don't want to answer, just let me know and I will go on to the next question. You can stop the interview at any time. In case you need more information about the survey, you may contact the person listed on the consent form and the information sheet. Thank you.   \|  \| **Identification** \| \| \| \| \| \| \| --- \| --- \| --- \| --- \| --- \| --- \| --- \| \| A \| Date of the interview \| \| \| \| \| \| \| B \| Name of the interviewer \| \| \| \| \| \| \| C \| Name of the district \| \| \| \| \| \| \| D \| Name of the clinic , health center, hospital \| \| \| \| \| \| \| E \| Language used for interview: \| \| \| \| \| \| \| F \| Anyone one present during interview?  Yes \| \| No \| If yes who: …………………………… \| \| \| \| G \| Data collection method  Self-administered \| \| \| - Interview \| \| - Both \| \| H \| Remarks by interviewer: \| \| \| \| \| \| \| I \| Consent form signed: \| - Yes \| \| \| - No \| \| \| J \| Data cross checked by: \| \| \| \| \| \|   **SECTION 1**  **Socio-demographic Characteristics**  *The following questions ask about your living situations. Please choose the option that best relates to you. Using a tick((√ )*   \| **No** \| **Question** \| \| **Response** \| \| --- \| --- \| --- \| --- \| \| SES 1 \| What is your place of residence? \| \| - Rural - Urban \| \| SES 2 \| What is your age (in years) \| \| - 1. Years - Don’t know \| \| SES 3 \| What is your current marital status? \| \| - Widowed - Divorced - In a relationship, living with partner - In a relationship, not living with partner - Single, never married, no current partner \| \| SES4 \| Is your partner HIV positive \| \| - Yes - No \| \| SES5 \| If yes: Is your spouse taking antiretroviral? \| \| - Yes - No \| \| SES 6 \| Do you have co-wife or wives \| \| - Yes - . No \| \| SES7 \| How many children do you currently have? \| \| ………………………………………….. \| \| SES8 \| What is your religion? \| \| - Protestant - Catholic - Born Again Christian - Adventist - Moslem - Traditional - Other (Specify) ………………………………… \| \| SES9 \| What is your educational status? \| \| - Never been to school - Primary - Secondary - O Level - Higher Secondary -A Level - Diploma - University Degree or Higher - Don’t know \| \| SES11 \| What is the educational status of your partner? \| \| - Never been to school - Primary - Secondary - O Level - Higher Secondary - A Level - Diploma - University Degree or Higher - Don’t know \| \| SES12 \| What is your occupation? \| \| - Student - Private business - Government / White collar job - Housewife - Daily laborer - Farmer - Other (specify)____________ \| \| SES13 \| What is the occupation of your spouse/partner? \| \| - Student - Private business - Government / White collar job - Housewife - Daily laborer - Farmer - Other (specify)____________ \| \| SES14 \| What is your monthly income in? \| \| Below   - 10,000 UGX - 10,001 – 100,000 UGX - 100,001 – 200,000 UGX - 200,001 – 300,000 UGX - 300,001 – 400,000 UGX - 400,001 – 500,000 UGX - 500,001 - Million UGX - I Million and above UGX \| \| SES 15 \| How would you describe your financial situation? \| \| - Poor or not enough - Enough - More than enough or well to do \| \| SES16 \| Do you have radio in your home? \| \| - Yes - No \| \| SES 17 \| Do you have TV in your home? \| \| - Yes - No \| \| SES 18 \| Do you have electricity in your home? \| \| - Yes - No \| \| SES 19 \| Do you have solar in your home? \| \| - Yes - No \| \| SES 20 \| Do you have a car in your home? \| \| - Yes - No \| \| SES 22 \| Do you have a refrigerator? \| \| - Yes - No \| \| SES 23 \| Do you have access to clean water? \| \| - Yes - No \| \| SES 24 \| Do you have access to toilet? \| \| - Yes - No \| \| SES 25 \| Do you have access to a pit latrine at home? \| - Yes - No \| \|   **SECTION 2**  **Health**  *The following items ask about the healthcare delivery system and how it relates to you. Please choose the option that best corresponds to you.*   \| **No** \| **Questions** \| **Response** \| \| --- \| --- \| --- \| \| HLT 1 \| What is the date you were diagnosed with HIV? (mm/did/yyyy) \| _____/ _____/ _____   - don’t remember \| \| HLT2 \| What date did you start antiretroviral treatment? (mm/dd/yyyy) \| _____/ _____/ _____   - Don’t remember \| \| HLT 3 \| How far do you live from the health facility? \| Km \| \| HLT 4 \| Where do you currently get your antiretroviral drugs? \| - At the antenatal clinic - At general antiretroviral treatment center - At pharmacy - At the health center \| \| HLT 5 \| Are you expected to pay for the antiretroviral drugs at the above facility \| - Yes - No \| \| HLT 6 \| If yes, do you find it difficult to make this payment \| - Yes - No \| \| HLT 7 \| Did your health care provider tell you how to take the medications? \| - Yes - No \| \| HLT 8 \| Did the health care provider counsel you on the common side effects of antiretroviral drugs? \| - Yes - No \| \| HLT 9 \| Do you have fears of taking the antiretroviral drugs? \| - Yes - No - Sometimes \| \| HLT 10 \| Did the health care provider tell you when to come back to the facility for your next appointment? \| - Yes - No \| \| HLT 11 \| When was your first antenatal visit? \| - First trimester (1-12 weeks) - Second trimester (13-28 weeks) - Third trimester (29-40 weeks) - Don’t know \| \| HLT 12 \| Do you have any previous elimination of mother-to-child transmission of HIV experience? \| - Yes - No \| \| HLT 13 \| Is your spouse/partner HIV positive? \| - Yes - No - Never tested - Don’t know \| \| HLT 14 \| If yes: Is your spouse taking antiretroviral? \| - Yes - No \| \| HLT 15 \| If divorced or separated, was the divorce /separation due to HIV? \| - Yes - No - I am living with my partner \| \| HLT 16 \| Did your doctor ever tell you that you have high blood pressure (hypertension) \| - Yes - No \| \| HLT 17 \| Did your doctor ever tell you that you have diabetes \| - Yes - No \| \| HLT 18 \| Did you doctor ever tell you that you have cancer \| - Yes - No \| \| HLT 19 \| Did your doctor ever tell you that you have tuberculosis (TB) \| - Yes - No \|   **SECTION 3**  **Center for Epidemiologic Studies Depression Scale (CES-D), NIMH**  *Below is a list of the ways you might have felt or behaved. Please tell me how often you have felt this way during the past week or tick (√ ) the number that best describe how you have felt or behaved.* | | | | | |
| --- | --- | --- | --- | --- | --- | --- | --- | --- | --- | --- | --- | --- | --- | --- | --- | --- | --- | --- | --- | --- | --- | --- | --- | --- | --- | --- | --- | --- | --- | --- | --- | --- | --- | --- | --- | --- | --- | --- | --- | --- | --- | --- | --- | --- | --- | --- | --- | --- | --- | --- | --- | --- | --- | --- | --- | --- | --- | --- | --- | --- | --- | --- | --- | --- | --- | --- | --- | --- | --- | --- | --- | --- | --- | --- | --- | --- | --- | --- | --- | --- | --- | --- | --- | --- | --- | --- | --- | --- | --- | --- | --- | --- | --- | --- | --- | --- | --- | --- | --- | --- | --- | --- | --- | --- | --- | --- | --- | --- | --- | --- | --- | --- | --- | --- | --- | --- | --- | --- | --- | --- | --- | --- | --- | --- | --- | --- | --- | --- | --- | --- | --- | --- | --- | --- | --- | --- | --- | --- | --- | --- | --- | --- | --- | --- | --- | --- | --- | --- | --- | --- | --- | --- | --- | --- | --- | --- | --- | --- | --- | --- | --- | --- | --- | --- | --- | --- | --- | --- | --- | --- | --- | --- | --- | --- | --- | --- | --- | --- | --- | --- | --- | --- | --- | --- | --- | --- | --- | --- | --- | --- | --- | --- | --- | --- | --- | --- | --- | --- | --- | --- | --- | --- | --- | --- | --- | --- | --- | --- | --- | --- | --- | --- | --- | --- | --- | --- | --- | --- | --- | --- | --- | --- | --- | --- | --- | --- | --- | --- | --- | --- | --- | --- | --- | --- | --- | --- | --- | --- |
|  | **During the Past Week** | | | |  |
|  | **Rarely or none of the time (less than 1 day)** | **Some or a little of the time (1-2  days)** | **Occasionally or a moderate amount of time (3-4 days)** | **Most or all of the time (5-7 days)** |  |
| CESD 1. I was bothered by things that usually don’t bother me. | 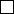 | 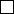 | 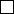 | 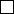 |  |
| CESD 2. I did not feel like eating; my appetite was poor. | 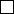 | 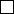 | 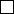 | 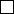 |  |
| CESD 3. I felt that I could not shake off the blues even with help from my family or friends. | 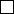 | 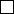 | 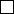 | 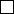 |  |
| CESD 4. I felt I was just as good as other people. | 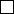 | 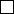 | 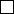 | 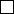 |  |
| CESD. I had trouble keeping my mind on what I was doing. | 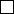 | 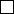 | 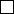 | 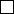 |  |
| CESD. I felt depressed. | 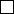 | 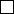 | 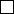 | 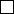 |  |
| CESD 7. I felt that everything I did was an effort. | 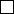 | 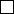 | 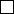 | 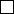 |  |
| CESD 8. I felt hopeful about the future. | 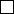 | 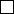 | 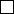 | 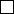 |  |
| DSE9. I thought my life had been a failure. | 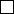 | 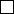 | 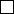 | 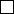 |  |
| CESD 10. I felt fearful. | 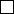 | 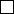 | 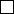 | 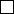 |  |
| CESD 11. My sleep was restless. | 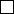 | 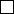 | 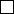 | 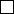 |  |
| CESD 12. I was happy. | 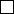 | 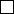 | 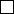 | 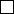 |  |
| CESD 13. I talked less than usual. | 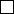 | 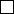 | 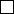 | 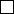 |  |
| CESD 14. I felt lonely. | 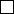 | 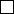 | 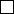 | 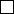 |  |
| CESD 15. People were unfriendly. | 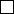 | 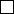 | 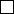 | 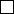 |  |
| CESD 16. I enjoyed life. | 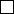 | 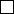 | 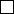 | 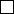 |  |
| CESD 17. I had crying spells. | 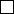 | 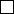 | 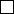 | 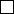 |  |
| CESD 18. I felt sad. | 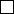 | 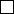 | 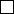 | 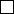 |  |
| CESD 19. I felt that people dislike me. | 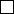 | 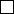 | 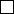 | 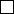 |  |
| CESD 20. I could not get “going.” | 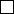 | 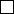 | 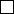 | 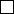 |  |
| **SECTION 4** | | | | | |

**Internalized Stigma of HIV/AIDS Tool (ISAT)**

*This section asks questions about your feeling about how you might have been feeling about your HIV status. Please tick (√ ) the number that best describe your situation based on the statement and how much you agree or disagree with each of them.*

| **No.** | **Item** | **Strongly disagree (1)** | **Disagree (2)** | **Neither agree nor disagree (3)** | **Agree (4)** | **Strongly agree (5)** |
| --- | --- | --- | --- | --- | --- | --- |
| ISAT 1 | Having HIV infection is like being branded with shame. |  |  |  |  |  |
| ISAT 2 | I feel blemished. |  |  |  |  |  |
| ISAT 3 | I feel ashamed about having  HIV/AIDS. |  |  |  |  |  |
| ISAT 4 | HIV infection hinders my ability to interact with other people |  |  |  |  |  |
| ISAT 5 | I feel that I need to hide my illness. |  |  |  |  |  |
| ISAT 6 | I try to hide that  I have HIV. |  |  |  |  |  |
| ISAT 7 | I feel that I am  desirable. |  |  |  |  |  |
| ISAT 8 | I feel inhibited from making new friends. |  |  |  |  |  |
| ISAT 9 | I am deceitful when I tell other people about my HIV. |  |  |  |  |  |
| ISAT 10 | HIV infection hinders me from being intimate with other people. |  |  |  |  |  |

**SECTION 5**

**Externalized Stigma of HIV/AIDS Tool (ESAT)**

*This section asks questions about your feeling about how you might have been treated because of your HIV status. Please tick (√ ) the number that best describe your situation based on the statement and how much you agree or disagree with each of them.* Have you ever experienced any of the following when you thought it was because of your HIV status?

| NO | Item | Strongly  Disagree | disagree | Neither agree or disagree | agree | Strongly agree |
| --- | --- | --- | --- | --- | --- | --- |
|  |  | (1) | (2) | (3) | (4) | (5) |
| ESAT 1 | Have you ever been excluded from social events? |  |  |  |  |  |
| ESAT 2 | Have you ever been abandoned by your spouse or partner? |  |  |  |  |  |
| ESAT 3 | Have you ever been abandoned by other family members? |  |  |  |  |  |
| ESAT 4 | Have you ever been told that it is your fault you have HIV? |  |  |  |  |  |
| ESAT5 | Have you ever been verbally abused or ridiculed? |  |  |  |  |  |
| ESAT 6 | Have you ever been physically assaulted by anyone? |  |  |  |  |  |
| ESAT 7 | Have you ever been fired from work or lost your job? |  |  |  |  |  |
| ESAT 8 | Have you ever been expelled from home? |  |  |  |  |  |
| ESAT 9 | Have you ever had property taken away? |  |  |  |  |  |
| ESAT 10 | Have you ever been denied health services? |  |  |  |  |  |

**SECTION 6**

**Interpersonal Support Evaluation List-12 (ISEL-12)**

*This section asks questions about your available interpersonal support. Please tick (√ ) the number that best describes your situation based on the statement and how much you agree or disagree with each of them.*

| **No** | **ISEL-12 item** | **Definitely true (3)** | **Probably true (2)** | **Probably false (1)** | **Definitely false (0)** |
| --- | --- | --- | --- | --- | --- |
| ISEL 1 | If I wanted to go on a trip for a day (for example to the beach, the country or mountains), I would have a hard time finding someone to go with me. |  |  |  |  |
| ISEL 2 | I feel that there is no one I can share my most private worries and fears with. |  |  |  |  |
| ISEL 3 | If I were sick, I could easily find someone to help me with my daily chores. |  |  |  |  |
| ISEL 4 | There is someone I can turn to for advice about handling problems with my family. |  |  |  |  |
| ISEL 5 | If I decide one afternoon that I would like to go to a movie that evening, I could easily find someone to go with me. |  |  |  |  |
| ISEL 6 | When I need suggestions on how to deal with a personal problem, I know someone I can turn to. |  |  |  |  |
| ISEL 7 | I don’t often get invited to do things with others. |  |  |  |  |
| ISEL 8 | If I had to go out of town for a few weeks, it would be difficult to find someone who would look after my house or apartment (the plants, pets, garden, etc.). |  |  |  |  |
| ISEL 9 | If I wanted to have lunch with someone, I could easily find someone to join me. |  |  |  |  |
| ISEL 10 | If I was stranded 10 miles from home, there is someone I could call who could come and get me. |  |  |  |  |
| ISEL 11 | If a family crisis arose, it would be difficult to find someone who could give me good advice about how to handle it. |  |  |  |  |
| ISEL 12 | If I needed some help in moving to a new house or apartment, I would have a hard time finding someone to help me. |  |  |  |  |

**SECTION 7**

**Case Adherence Index**

*This section asks questions about your feelings about taking the ARVs as prescribed by your healthcare provider. How much do you agree or disagree with each of the following statements about adherence to the ARV. Tick (√) the number that best describe your feeling.*

ADH 1. How often do you feel that you have difficulty taking your HIV medications on time? By
‘on time’ we mean no more than two hours before or two hours after the time your doctor told
you to take it.

- Never
- Rarely
- Most of the time
- All of the time

ADH 2. On average, how many days per week would you say that you missed at least one dose of
your HIV medications?

- Everyday
- 4–6 days/week
- 2–3 days/week
- Once a week
- Less than once a week
- Never

ADH 3. When was the last time you missed at least one dose of you HIV medications?

- Within the past week
- 1–2 weeks ago
- 3–4 weeks ago
- Between 1 and 3 months ago
- More than 3 months ago
- Never

ADH 4: Are you still taking your ARV drugs every day?

- Yes
- No
- Not every day

ADH 5: After we talked with you the last time did you go to the hospital to collect your ARVs drug or for checkup

- Yes
- No
- Never

ADH 6: When was the last time you visited hospital to collect your ARV drug or for checkup?

- Less than one month
- Between one and two months
- More than two months ago
- I do not remember

**SECTION 8**

**Household Food Insecurity Access Scale (HFIAS)**

*This next section asks questions about the situation of food availability in your household. Please tick (√) the number that best describe your household situation and how much you agree or disagree with each of the following questions.*

HFIAS 1. In the past four weeks, did you worry that your household would not have enough food?

- 0 = No (skip to Q2)
- 1=Yes

HFIAS 1 A. How often did this happen?

- 1 = Rarely (once or twice in the past four weeks)
- 2 = Sometimes (three to ten times in the past four weeks)
- 3 = Often (more than ten times in the past four weeks)
- I don’t know

HFIAS 2. In the past four weeks, were you or any household member not able to eat the kinds of foods you preferred because of a lack of resources?

- 0 = No (skip to Q3)
- 1=Yes

HFIAS 2A. How often did this happen?

- 1 = Rarely (once or twice in the past four weeks)
- 2 = Sometimes (three to ten times in the past four weeks)
- 3 = Often (more than ten times in the past four weeks)
- 0=Declined to answer

3. In the past four weeks, did you or any household member have to eat a limited variety of foods due to a lack of resources?

- 0 = No (skip to Q4)
- 1=Yes

HFIAS 3A. How often did this happen?

- 1 = Rarely (once or twice in the past four weeks)
- 2 = Sometimes (three to ten times in the past four weeks)
- 3 = Often (more than ten times in the past four weeks)
- 0=Declined to answer

HFIAS 4. In the past four weeks, did you or any household member have to eat some foods that you really did not want to eat because of a lack of resources to obtain other types of food?

- 0 = No (skip to Q5)
- 1=Yes
- 0=Declined to answer

HFIAS 4A. How often did this happen?

- 1 = Rarely (once or twice in the past four weeks)
- 2 = Sometimes (three to ten times in the past four weeks)
- 3 = Often (more than ten times in the past four weeks)
- 0= I don’t remember

HFIAS 5.In the past four weeks, did you or any household member have to eat a smaller meal than you felt you needed because there was not enough food?

- 0 = No (skip to Q6)
- 1=Yes

HFIAS 5A. How often did this happen?

- 1= Rarely (once or twice in the past four weeks)
- 2= Sometimes (three to ten times in the past four weeks)
- 3= Often (more than ten times in the past four weeks)
- 0=Declined to answer

HFIAS 6. In the past four weeks, did you or any other household member have to eat fewer meals in a day because there was not enough food?

- 0 = No (skip to Q7)
- 1=Yes

HFIAS 6A. How often did this happen?

- 1= Rarely (once or twice in the past four weeks)
- 2= Sometimes (three to ten times in the past four weeks)
- 3= Often (more than ten times in the past four weeks)

HFIAS 7. In the past four weeks, was there ever no food to eat of any kind in your household because of lack of resources to get food?

- 0 = No (skip to Q8)
- 1=Yes

HFIAS 7A. How often did this happen?

- 1= Rarely (once or twice in the past four weeks)
- 2= Sometimes (three to ten times in the past four weeks)
- 3= Often (more than ten times in the past four weeks)

HFIAS 8. In the past four weeks, did you or any household member go to sleep at night hungry because there was not enough food?

- 0= No (skip to Q9)
- 1=Yes

HFIAS 8A. How often did this happen?

- 1= Rarely (once or twice in the past four weeks)
- 2= Sometimes (three to ten times in the past four weeks)
- 3= Often (more than ten times in the past four weeks)

HFIAS 9. In the past four weeks, did you or any household member go a whole day and night without eating anything because there was not enough food?

- 0 = No
- 1=Yes

HFIAS 9A. How often did this happen?

- 1= Rarely (once or twice in the past four weeks)
- 2= Sometimes (three to ten times in the past four weeks)
- 3= Often (more than ten times in the past four weeks)
